# NetControl4BioMed: a web-based platform for controllability analysis of protein–protein interaction networks

**DOI:** 10.1093/bioinformatics/btab570

**Published:** 2021-08-05

**Authors:** Victor-Bogdan Popescu, José Ángel Sánchez-Martín, Daniela Schacherer, Sadra Safadoust, Negin Majidi, Andrei Andronescu, Alexandru Nedea, Diana Ion, Eduard Mititelu, Eugen Czeizler, Ion Petre

**Affiliations:** Computer Science Unit, Åbo Akademi University, Turku 20520, Finland; Department of Information Systems, Polytechnic University of Madrid, 28040 Madrid, Spain; Faculty of Mathematics and Computer Science, Heidelberg University, 69120 Heidelberg, Germany; Department of Computer Science and Engineering, Koç University, Rumelifeneri Yolu 34450 Sarıyer, İstanbul, Turkey; Department of Computer Science and Engineering, University of California, Santa Cruz, CA 95064, USA; Faculty of Electronics, Telecommunications and Information Technology, POLITEHNICA University of Bucharest 061071, Romania; Faculty of Electronics, Telecommunications and Information Technology, POLITEHNICA University of Bucharest 061071, Romania; Faculty of Electronics, Telecommunications and Information Technology, POLITEHNICA University of Bucharest 061071, Romania; Faculty of Electronics, Telecommunications and Information Technology, POLITEHNICA University of Bucharest 061071, Romania; Computer Science Unit, Åbo Akademi University, Turku 20520, Finland; Department of Bioinformatics and Biostatistics, National Institute for Research and Development for Biological Sciences, Bucharest 060031, Romania; Department of Bioinformatics and Biostatistics, National Institute for Research and Development for Biological Sciences, Bucharest 060031, Romania; Department of Mathematics and Statistics, University of Turku, Turku 20014, Finland

## Abstract

**Motivation:**

There is an increasing amount of data coming from genome-wide studies identifying disease-specific survivability-essential proteins and host factors critical to a cell becoming infected. Targeting such proteins has a strong potential for targeted, precision therapies. Typically however, too few of them are drug targetable. An alternative approach is to influence them through drug targetable proteins upstream of them. Structural target network controllability is a suitable solution to this problem. It aims to discover suitable source nodes (e.g. drug targetable proteins) in a directed interaction network that can control (through a suitable set of input functions) a desired set of targets.

**Results:**

We introduce NetControl4BioMed, a free open-source web-based application that allows users to generate or upload directed protein–protein interaction networks and to perform target structural network controllability analyses on them. The analyses can be customized to focus the search on drug targetable source nodes, thus providing drug therapeutic suggestions. The application integrates protein data from HGNC, Ensemble, UniProt, NCBI and InnateDB, directed interaction data from InnateDB, Omnipath and SIGNOR, cell-line data from COLT and DepMap, and drug–target data from DrugBank.

**Availabilityand implementation:**

The application and data are available online at https://netcontrol.combio.org/. The source code is available at https://github.com/Vilksar/NetControl4BioMed under an MIT license.

## 1 Introduction

Genome-wide association studies led in the last few years to an increasing availability of data on disease-specific survivability-essential genes (Koh *et al.*, 2012) and on host factors critical to cell infection (Daniloski *et al.*, 2021). Such data can be used in network-based drug repurposing studies (Morselli Gysi *et al.*, 2021). The concept is to trace the cascading signals of drug combinations through directed protein–protein interactions from the drug targets to the essential/critical proteins. One of the promising computational approaches to this problem is target network controllability, that can be used to identify combinations of drug targetable proteins controlling a set of critical targets in a directed network. Several formulations and demonstrations of this approach exist, especially on Boolean network controllability (Biane *et al.*, 2019; Murrugarra *et al.*, 2016; Zañudo *et al.*, 2015) and on target structural controllability (Kanhaiya *et al.*, 2017; Wei-Feng *et al.*, 2017).

We introduce NetControl4BioMed, a free open-source web-based software, aimed at applications in biomedicine and allowing for: (i) constructing directed protein–protein interaction networks, (ii) structural target network controllability analysis focused on identifying effective drug-combinations and (iii) sharing networks and analyses between users. It is a re-engineering of the first version of the software (Kanhaiya *et al.*, 2019), from the algorithms and the implementation to the interface and the functionality. NetControl4BioMed allows multi-user collaborative access to building directed protein–protein interaction networks around a set of seed proteins of interest, to upload such networks from external platforms, and to perform structural target controllability analyses with a focus on drug combination identification. Several approaches exist for Boolean network controllability (Biane *et al.*, 2019; Lin *et al.*, 2012; Murrugarra *et al.*, 2016; Su *et al.*, 2021; Zañudo *et al.*, 2015). For structural target controllability the only other tool that we are aware of is the Cytoscape app CytoCtrlAnalyser (Wu *et al.*, 2018). In comparison to CytoCtrlAnalyser, NetControl4BioMed offers the ability to generate directed protein–protein interaction networks, a much more customizable search, integration with multiple external databases, including drug data and several cell line gene essentiality data, a cloud-based approach to network analysis, independent of the performance of the user’s own system, and the possibility for multi-user collaboration.

We discuss in the next sections the data that NetControl4BioMed integrates and its usability for network generation and network analysis.

## 2 Data

We use pre-compiled protein data from the public online HGNC (Braschi *et al.*, 2019), Ensembl (Yates *et al.*, 2019), UniProt (Consortium, 2021), NCBI (Brown *et al.*, 2015) and InnateDB (Breuer *et al.*, 2013) databases, with all the corresponding unique identifiers being integrated by the application. The interaction data uses experimentally validated information from the Omnipath (Türei *et al.*, 2016), InnateDB (Breuer *et al.*, 2013) and SIGNOR (Licata *et al.*, 2020) databases. The data contains 42 152 proteins and 46 942 interactions. The application also provides a set of 1578 pre-compiled protein collections, consisting of 52 sets of disease-specific survivability-essential genes for several cancer cell-lines from COLT (Koh *et al.*, 2012), the 1526 sets of mutated genes for several cancer cell-lines from DepMap (Boehm *et al.*, 2021), and the 9 sets of drug–target genes from DrugBank (Wishart *et al.*, 2018).

## 3 Network generation

To generate a network the user needs to specify the following: (i) the list of seed protein identifiers around which the network will be built, (ii) the interaction database(s) to be used by the network and (iii) the algorithm for the network generation. Several algorithms are available: selecting all interactions containing the seed proteins, selecting only direct interactions between the seed proteins, selecting the interactions between seed proteins with at most one to four intermediary proteins. The output consists of a network which can be inspected, downloaded for external use and visualization, or used further in the application for analysis. The size of the generated networks varies based on the number of seed proteins, the number of selected interaction databases and the generation algorithm. Networks with tens of thousands of interactions can easily be handled by the software.

## 4 Network analysis

To run a controllability analysis the user needs to specify the following: (i) the network to be analyzed, (ii) (optional) the list of source protein identifiers which would be preferred as control inputs, (iii) the list of target protein identifiers which should be controlled and (iv) the algorithm for the controllability analysis and its parameters. Two controllability algorithms are available: the greedy algorithm described in Czeizler *et al.* (2018) and the genetic algorithm described in Popescu *et al.* (2021). Each algorithm requires several specific parameters, and predefined default values for each parameter are available. The output of the analysis consists of one or more sets of control paths, each of them containing the list of control inputs able to control the entire target set (with the drug–targets among them distinctly marked), as well as the list of individual paths between each target and its corresponding control input. These control paths can be individually inspected and downloaded for external use and visualization. The duration of the controllability analysis varies based on the size of the network, the number of target proteins and the parameters of the algorithm. The analysis runs on the server and the user is notified when the results are available.

## 5 Conclusions

We present a new web application for network generation and network structural target controllability analysis, with a focus on biomedicine. The software provides a modern and friendly user interface, allowing for sharing and collaboration between users. We provide several already compiled and ready-to-be-used datasets on protein–protein interaction networks, disease-specific survivability-essential and mutated genes and drug–target genes. We believe that the application will facilitate experimenting and effective application of network analysis techniques in the biomedical domain. It can be potentially useful to researchers for better understanding of interaction networks pathway structure, for identifying novel therapeutic suggestions, and for a patient- and disease-specific personalized approach to treatment.

## Funding

This work was partially supported by the Romanian Ministry of Education and Research, CCCDI--UEFISCDI (project number PNIII-P2-2.1-PED-2019-2391, within PNCDI III awarded to IP) and by the Academy of Finland (project number 311371 awarded to EC).

*Conflict of Interest*: none declared. 
